# Mothers’ and father’s perceptions of the risks and benefits of screen time and physical activity during early childhood: a qualitative study

**DOI:** 10.1186/s12889-018-6199-6

**Published:** 2018-11-20

**Authors:** Trina Hinkley, Jennifer R. McCann

**Affiliations:** Deakin University, Institute for Physical Activity and Nutrition (IPAN), School of Exercise and Nutrition Sciences, 221 Burwood Highway, Burwood, VIC 3125 Australia

**Keywords:** Early childhood, Physical activity, Active play, Parents, Perceptions, Screen time

## Abstract

**Background:**

This study sought to explore mothers’ and fathers’ perceptions of the risks and benefits of screen time and active play during early childhood.

**Methods:**

In-depth semi structured telephone interviews were conducted with mothers and fathers (*n* = 28) of children aged 3–5 years who had earlier taken part in a larger quantitative study in Australia and identified willingness to be re-contacted were recruited. Interviews were audio recorded, transcribed verbatim, and analysed using NviVo. Coding was performed to produce themes. Quotes were extracted from the transcripts to illustrate common responses. COREQ guidelines for qualitative papers were followed.

**Results:**

Parents reported active play was beneficial for many health and developmental outcomes such as imagination, enjoyment and socialisation, while reporting risks such as safety and stranger danger. There were mixed perceptions of screen time, with benefits such as learning, education and relaxation, and risks including habit formation, inappropriate content, negative cognitive and social outcome, and detriments to health being reported. A few differences between mothers’ and fathers’ perceptions were evident.

**Conclusion:**

This study identified that some parental perceptions of benefits and risks of screen time and active play were consistent with published evidence, while others were contradicted by current evidence. Future studies should consider evidence-based education to ensure parents are aware of evidence-based outcomes of children’s behaviours. Interventions may wish to capitalise on parents perceived benefits to enhance engagement.

**Electronic supplementary material:**

The online version of this article (10.1186/s12889-018-6199-6) contains supplementary material, which is available to authorized users.

## Background

Early childhood is a crucial developmental period when children lay the foundation for health and development [1]. This is also a critical period for brain development [2]. Two behaviours which contribute towards children’s health and development are physical activity and screen time. Evidence is accumulating which suggests that preschool children’s screen time may be detrimental to their physical, [3] psychosocial [4] and cognitive [5] development while physical activity may support beneficial outcomes across all areas of development [4, 6, 7]. Despite this, studies report that children participate in high levels of screen time and low levels of physical activity during this period [8, 9].

As a consequence of potential benefits to health and development, and low levels of compliance with guidelines, several interventions targeting behaviour change have been implemented over the past decade [10, 11]. Recent systematic reviews of those interventions suggest that these behaviours may be resistant to change: fewer than half of identified studies reporting on interventions to reduce screen time or increase physical activity during early childhood were found to be effective [10, 11] despite a growing body of evidence on correlates of these behaviours to target in such interventions [12–15]. Thus, further consideration of parents’ perceptions of the behaviours is warranted.

Several studies have reported parents’ apparent lack of concern regarding children’s levels of physical activity and screen time [16, 17], including the perception that young children are naturally physically active [18]. Thus, interventions directly focusing on the behaviours themselves may not be engaging to parents. Nonetheless, screen time was recently identified as Australian parents’ number one concern with respect to children’s health, [19] suggesting that there may be growing awareness of potential adverse impacts of screen time.

Little is known about how parents perceive either the behaviours themselves, or the potential impact of these behaviours on their children’s health and development, during the early childhood period. Studies investigating parents’ perceptions of sedentary behaviour and physical activity guidelines in Canada and the United Kingdom, and one study exploring broader aspects of physical activity and screen time in Australian children, have provided some insights [18, 20, 21]. While some parents report educational, cognitive [20] and social [21] benefits from screen time, most recognise that interaction with a care-giver provides the most appropriate opportunities for learning. [20] Some parents also report awareness of the potential for screen time to adversely affect their child in other ways (e.g. addiction, unresponsiveness) [18, 21]. However, a thorough exploration of parents’ perceptions of the impact of the behaviours is lacking, as is exploration of Australian parents’ perceptions of current guidelines. Such data are necessary to provide essential insight to inform effective behaviour change strategies. The Health Belief Model [22] may be useful to explain how parents’ perceptions of the potential risks from screen time for their young child’s health and development might consequently impact children’s actual screen time and active play behaviors, as well as to explore whether mothers and fathers have different beliefs about risks in relation to screen time and their young children. There is no existing exploration as to whether parental perceptions vary by parent sex, as the majority of studies with children in this age group include only or primarily mothers [23–25]. Identifying if differences exist may further enhance intervention strategies with respect to how mothers and fathers are engaged and encouraged to maintain participation in the intervention. Additionally, there is a lack of published evidence of the strategies which parents perceive may be beneficial in supporting improvements in health behaviours.

The aims of this paper are to explore parents’ perceptions of various aspects of screen time and active play in their children including:

1. the risks and benefits of each of the behaviours;
2. awareness and acceptance of existing Australian guidelines;
3. potential strategies which may support behaviour change; and
4. potential differences between the mothers’ and fathers’ perceptions.

## Methods

### Design, setting and participants

Parents were recruited from a larger study which was conducted to develop an understanding of physical activity and screen behaviours of parents and their young children. Recruitment for the main study occurred through kindergartens, childcare centres, playgroups as well as parenting websites and blogs. Contact was initially made through email, with study flyers emailed to the contact person, who then disseminated them to the parents either through email or hard copies, or in the case of websites and blogs, by inclusion of the study details in their writing or web posts. In total, 756 parents provided complete data in the main study (586 mothers and 170 fathers). Details have previously been published [26, 27].

At the conclusion of data collection for the main study, parents were asked to provide contact details if they wished to be contacted for participation in future research. Participants in this study were drawn from those who indicated they would be willing to be re-contacted. In total 467 parents (369 mothers and 98 fathers) consented to being contacted for future research. All participants had to have a child between the ages of 2–5 years old, and live in Australia to take part. The list of eligible participants was randomly ordered and 53 mothers and 14 fathers were emailed a maximum of two times with an invitation to participate. Those participants that responded (18 mothers and 13 fathers, from independent families) were then followed up with a phone call to schedule an interview time. In total, 16 mothers and 12 fathers were interviewed as the remainder were cancelled by the parent.

### Data collection

Semi-structured interviews, with follow up questions and probes, were conducted. While the interview schedule was not strict in nature, the questions were asked in largely the same sequence to aid in data processing and interviewer schedule prompts. Interviews were conducted over the phone in June and July 2014 and were audio-recorded.

In summary, the interview questions focused on parent’s perception of the risks and benefits of physical activity (operationalised as active play) and screen time, knowledge of any guidelines for each, and finally strategies to change behaviours relating to increasing physical activity and decreasing screen time (Additional file 1).

Participants provided verbal consent to participate in the study as well as consent for the interview to be audio recorded at the beginning of the interview. Active play (“all those times your child is doing something active such as playing outside or running around the house”) and screen time (“anything and everything which has an electronic screen: TV/video/DVD viewing, computer and electronic game use and smart phone or digital tablet use”) were explained to all participants, so they could apply the definitions when answering context-specific questions relating to each. Twenty-eight interviews were conducted in total. Interviews lasted between 17 and 59 min (mean 30 min, median 28 min). Demographic data were collected from the main parent survey previously completed.

Data collection ceased once data saturation was reached and no new themes were emerging from the interviews. Ethics approval was provided by the Deakin University Human Ethics Advisory Group-Health HEAG-H 87_2014, as well as from the Department of Education and Early Childhood Development 2013_002144.

### Data analysis

Descriptive characteristics for demographic data were calculated to describe the sample. All interviews were audio recorded and transcribed verbatim by a transcription company. Data analysis using NviVo (V.10.0 QSR, Southport, UK) began once the interview transcripts were received back from the transcription company. One researcher conducted all the coding and met with the senior researcher to discuss the results and confirm codings. Once common themes were identified for each area of investigation, quotes were extracted from the transcripts and analysed for appropriateness as structured question-discussion/answer format.

## Results

Participant characteristics are displayed in Table 1. More mothers than fathers participated; all participants were currently living in Australia, most in Victoria. All mothers reported being responsible for caring for their child between 50 and 100% of the time, while fathers ranged from less than 25 to 75% of the time.

**Table 1:** Participant characteristics

Participant characteristics	
Child age (years; mean (SD))	3.7 (1.1)
Child sex (n (%))	
Boys	16 (57)
Girls	12 (43)
Parent age (years; mean (SD))	
Fathers	40 (4)
Mothers	36 (5)
Parent sex (n (%))	
Fathers	12 (43)
Mothers	16 (57)
Marital status (n (%))	
Married	25 (89)
Separated	3 (11)
Parental education (n (%))	
Year 12 or lower	1 (3.6)
Trade	4 (14.3)
Diploma	2 (7.1)
Undergraduate degree	10 (35.7)
Postgraduate	11 (39.3)
Geographic location (within Australia) (n (%))	
Victoria	18 (64)
New South Wales	3 (11)
Queensland	4 (14)
Western Australia	2 (7)
Australian Capital Territory	1 (4)
Child’s school status (n (%))	
Started school	5 (18)
Not started school	23 (82)
Percent of time parent is primary carer (n (%))	
< 25%	12 (43)
Fathers	6 (21)
Mothers	0
25–50%	3 (11)
Fathers	3 (11)
Mothers	0
50–75%	7 (25)
Fathers	3 (11)
Mothers	4 (14)
> 75%	12 (43)
Fathers	0
Mothers	12 (43)

### Parental perceptions of the risks and benefits of active play

All parents reported that active play was either equally important with screen time or very important in its own right, in regards to their child. As one dad put it *“I think he’s better off out there playing than he is sitting in front of the television. It’s better for him in terms of his development in that area in my opinion” (20509).* Parents identified a range of benefits of active play. Most parents (68%) commented on the health and developmental outcomes of active play, which primarily focused on mental health, supporting the immune system and cognitive development. As one mother said, *“It really helps in his coordination, he’s got a better appetite when he’s been more active. Kids need a bit of Vitamin D. He is able to concentrate better, and it is not just all about the physical benefits, it has mental benefits as well” (10282).* Similarly, another mother noted, *“They play in dirt which is good for their immune system, they are outside and breathing the oxygen and so their body is getting a lot of good stuff” (10835).* One father commented *“a huge part of it is brain development and what they’re getting through exploring themselves, and exploring the world around them” (20649).*

Approximately half of all parents (48%) reported that active play provided opportunities to foster imagination and enjoyment. For example, one mother said, *“I really like fostering his imagination with active play” (10179),* while one father stated that active play was *“for fun, to show her that play can be fun, as a way of us doing things together” (20832).*

Many parents (36%) also mentioned the social benefits of active play. For instance, one mother said, *“When they’re playing, they’re playing with other children. They’re often playing games and it teaches them fairness and sociability and sharing and all those things” (10542).* Another reflected, “*a lot of social interaction goes on as part of that, too. To me that’s all building on those key life skills that he’s going to need down the track…” (10589).*

Parents’ concerns about active play mainly focused on safety issues, including potential for injury (57%). While some parents were worried about the need to supervise their child/ren in an effort to prevent injury, others reported that injuries were part of growing up. One mother commented, *“he needs constant supervision. So things like jumping on the bed, and building cubby houses on the couch, that’s all really good, and it’s all using up activity, but you need to be there to supervise” (10179).* A father had this to say about stranger danger, *“She will either walk up or swim up to people and talk to them, like random strangers. As a parent that can freak you out a little bit” (200028).* One mother summed it up nicely, *“there’s risk in everything that you do but I’d much rather him fall over and hurt himself than never go outside” (10386).*

Very few parents appeared to be concerned about their child undertaking too much active play (7%). However, those who were concerned perceived the potential for too much active play to have negative outcomes on sleep. For instance, one mother remarked, *“She does too much some days, because I get concerned about her being overtired and then she might not sleep so well” (10880).*

### Parental perceptions of the risks and benefits of screen time

Some parents (25%) stated that screen time was not important at all in their home. For instance, one mother commented ***“****I’m an advocate for children not having any sort of electronic media and I try and push that myself through my own work” (10835).* However, an equal number (25%) believed that screen time was either independently important, or equally important, with active play. Parents who held these views primarily believed that it was important to give children opportunities to learn how to use technology, as this mother states: ***“****I do think that it’s going to be important that he does know how to use a computer; by the time he reaches school he’ll probably be doing a lot of computer-based learning”* (10341).

The most common perceived benefit was that screen time provided opportunities for learning and education (stated by 82% of parents), as illustrated by this mother: ***“****Other benefits are also things that he learns, like vocabulary, sometimes he comes out with words and I think, ‘Wow, where did he get that from?’ Or phrases, or the way that he says things. And I can actually relate it back to something that he’s watched” (10179).* Similarly, a number of parents (18%) perceived that screen time improved cognitive outcomes in their children, as mentioned by this mother “*educational games, and getting him to read and write, doing interactive (online) books that help him to recognise words and do matching colours and those sorts of things” (10282).*

Half of all parents interviewed believed screen time was important for relaxation. As this father stated *“it’s a good way for her to veg out, because we don’t necessarily want her to sleep because then it’ll disrupt her night sleep patterns, so it’s a good way for her to have a rest and some downtime” (20832).* Screen time was also reported to be useful as a babysitter to allow other responsibilities to be fulfilled or for parents to take some time out (32% of parents reported this). For instance, one mother stated: *“we are happy that he goes and sits in front of the TV some afternoons … because it means that we can go do what we need to do, or we can sit down and have a break for five minutes” (10341).*

Only a very small number of parents (7%) stated that they believe that screen time provided no benefit at all for their young children. For instance these comments typify what fathers and mothers, respectively, reported: ***“****I don’t see it as a beneficial activity.” (20614);* and *“At this age, I don’t really see a lot of benefits but I think they come with time” (10542).*

Many parents (79%) identified a number of potential risks of screen time. For instance, several parents (32%) were concerned about the risk involved in screen time becoming a habitual behaviour. As one mother commented: ***“****I don’t want it to become a habit. I don’t want him to be a teenager that’s going to sit in front of the TV all day” (10341).* A number of parents (29%) perceived potential for screen time to impact their child’s physical health, as stated by this father: ***“****The posture that they have when they’re sitting playing the games on couches, you know, their necks are down, they’re focussing really intently. So it’s going to obviously affect their necks, their shoulders and eye sight” (20638).* Several parents (29%) also perceived potential for their children to view inappropriate comment, as stated by this father: ***“****He may see something that is potentially harmful to him or that he’s not ready to process emotionally or cognitively” (20100).*

The majority (79%) of parents raised at least some concerns, with many (32%) worrying about the ‘mind numbing’, or ‘zoning out’ nature of screen time. This father commented: “*He loves the idea of watching the television and we try and limit it, because the more he watches the more he wants to watch it, the more he asks and we realise he’s watching too much” (20278).* Similarly, this mother was concerned: *“He’s not using his brain” (10341).*

Negative cognitive and social outcomes were also mentioned by several parents (25%), with one mother commenting: “*if it’s not controlled within certain limits, then it might impact negatively on development, you know in development of language, and movement, co-ordination and that kind of stuff, because there’s not enough practice of those things” (10614).* Another mother was concerned about the impact of her child’s screen time on brain development and attention: “… *they’re in that developing stage. Neurons are developing in their brains, its wiring them how they’re not supposed to be wired naturally and I think that it is having an impact on children’s behaviour and their learning. Attention deficit disorder, learning difficulties, are so much more prevalent now, I believe that’s affecting it” (10835).* One father commented on the anti-social aspect of screen time: “*it becomes very unsociable, especially if we’re going out, and they want to take their iPads with them” (20638).* Similarly, this mother commented: *“because I think there are a lot of other activities that kids can be doing and I can’t help but feel that for say, two hours it means that’s being used more as a babysitter rather than anything else. I think that social interaction is important as well (10589).”*

### Awareness and acceptability of guidelines

Overall, 15% of parents could accurately report current Australian guidelines for both physical activity and screen time for their child. A further 68 and 18% of parents were aware that screen time and physical activity, respectively, guidelines existed but could not state what they were. Typical responses included those like this father’s when asked if he knew what the Australian recommendation was: ***“****No, not really, just kids should be active. Not watch too much TV” (20405).* Another father identified confusion about current guidelines as follows: ***“****I’ve seen all sorts of different things. I’ve seen stuff that says no screen time for kids under two, or even stuff that says no screen time for kids under six” (20614).*

Despite a lack of awareness about what the guidelines were, most parents (60%) agreed that guidelines were important. However, parents also reflected that individual practices within their family were what they believed mattered most. For instance, one father had this to say, ***“****whether the guidelines are there and us trying to follow the guidelines is less important than we are doing what we think is right and the guidelines seem to reflect what we do” (20278).* Other parents believed that characteristics of their own childhood were appropriate and should be similar for their children, such as this father: ***“****I never was allowed to watch that much television, so I don’t really believe they should be either” (20594).*

### Behaviour change strategies

Parents were very forthcoming with strategies to decrease screen time and increase active play. Almost half of all parents (43%), such as this father, believed that parental modelling of appropriate behaviour was essential: ***“****I don’t see how anyone could make a child do it if they weren’t prepared to do it themselves” (20638).* Additionally, several participants (39%) believed that parents needed to take control, set limits for their children and more closely monitor their child’s screen time. For instance, one mother believed “*that the parents can keep track of how much their kids have been watching, even like a little timer. You know how they’ve got those egg timers for the shower, you could have one for the TV” (10210).* One father suggested getting rid of the television altogether as a possible strategy to decrease screen time in the household, “*we could get rid of our TV, that would be the thing that would make it easiest to change our habits if we wanted to ‘cause then it’s just not there and you can’t watch it” (20405).*

Some parents (25%) believed media and advertising campaigns could be useful, although one mother questioned whether such a campaign would be sufficient. That mother believed that reputable information from a diversity of sources would stimulate potential need for change: *“I think if society was more aware of those type of recommendations and the reasons why those recommendations are there and that was coming from lots of different sources… that would definitely help me to think that or to do something about how much TV my kids watched” (10952).* Similarly, some parents (18%) mentioned dissemination of reputable research findings as a possible strategy, particularly coming from sources that parents had easy access to and respected such as schools and early childhood settings: *“coming from teachers and from researchers. Because I work in early childhood I have access to those but I don’t think that normal parents do. If it comes from the school, also I guess as a token if it comes from the media, that would be good. That would make an impact” (10835).* A few parents (7%) suggested that increasing neighbourhood play equipment may help support children to be more active.

### Differences between mothers and fathers

Although parents mostly reported similar perceptions and beliefs of children’s behaviours, some differences between mothers’ and fathers’ perceptions were evident. For instance, a higher proportion of mothers (25%) reported using screen time as a babysitter for their children compared with fathers (7%), while a higher proportion of fathers (21%) mentioned the health risks of screen time compared with mothers (7%).

## Discussion

This study has explored parental perceptions of physical activity and screen time and identified benefits, risks and awareness of guidelines. Notably, some parents were able to appropriately identify benefits of physical activity, and risks of screen time, which are consistent with published evidence. For instance, evidence suggests that physical activity is supportive of children’s movement skills, [7] attention [6] and cognitive development [6] and that screen time may be detrimental to children’s language development, attention and social interactions [5, 28]. Thus, a number of the identified benefits of physical activity and risks of screen time were consistent with published evidence.

However, parents also reported risks of physical activity and benefits of screen time which are not consistent with associations in the extant literature. Consistent with previous qualitative research, [16] parents’ concerns about physical activity were focused on injury and stranger danger. However, no research is available to show evidence of associations between level of injury or other adverse events as a consequence of physical activity during early child, nor even during the primary school years [7, 29]. Parents also identified a number of benefits of screen time which are inconsistent with published evidence. These included education and learning to use technology. While a small number of studies show that children may gain skills such as language and other educational benefits from screen time, [30, 31] generally such findings result from specific content from a small number of shows and fail to account for a broad range of cognitive outcomes or viewing contexts. Such associations are typically not found in studies exploring habitual screen time where inverse associations between behaviour and outcomes are evident [3, 6]. Thus it may be necessary to raise parental awareness of the impact of screen time on children’s cognitive outcomes as previous research has shown that parents who perceive that screen time adversely impacts children’s cognitive functioning have children who engage in lower levels of screen time [26].

The variety of benefits identified by parents in this study which are supported by quantitative evidence suggest that it may be possible to adopt alternative approaches to behaviour change rather than focusing on the behaviours themselves. Such approaches may include stealth approaches which make the process of change easy and desirable, and focus on an outcome of value, rather than the behaviour itself [32]. As an example, a previous intervention in adolescent girls focused on providing after school dance classes for girls; however, the primary outcome was reduction in weight status although this was not overtly promoted to participants [33]. Thus, the stealth approach focused on an activity – dance – to make the process of change – weight reduction – easy to engage in. That is, a stealth approach to changing physical activity or screen time could focus on children’s social skills or language development, incorporating strategies which target these outcomes directly and incorporate the behaviours of interest. Parents reported that they value the timely development of children’s social skills and language skills. Thus, targeting and promoting those in interventions, while incorporating activities to increase physical activity or reduce screen time, could effectively engage parents to participate in the intervention in the first place.

The low levels of awareness of the Australian guidelines for physical activity and screen time in this study are congruent with those reported in parents in Canada and the United Kingdom [18, 20]. Similar to the United Kingdom, little work has been undertaken by authorities in Australia to disseminate and publicise the guidelines for early childhood. Thus, the lack of awareness of guidelines in this sample of parents is unsurprising. It is clearly necessary to raise awareness of current guidelines for physical activity and screen time during early childhood in parents, along with the possible benefits of engaging in health levels of these behaviours. In addition, promoting these messages to key influencers of this target population, such as early childhood educators, health professionals and community health workers, will help to further raise awareness in the target population. Campaigns raising awareness of the guidelines themselves may be beneficial in eliciting improvements in children’s physical activity and screen time behaviours.

Parents identified a number of strategies which they believed may support changes in children’s behaviours which have previously been trialled in intervention studies such as setting limits, keeping track of screen time and even using mass media campaigns to disseminate this type of information [34]. This would suggest that parents believe these strategies have the potential to be effective. Of particular note, several parents mentioned the need for information to come from a variety of reputable sources, which may be key to effecting behaviour change in this population, with schools and early childhood centres being potential avenues through which information and strategies could be disseminated.

Finally, this study found that mothers and fathers held similar perceptions of their children’s behaviours. However, the contexts provided, and actual behaviours undertaken with their children, by mothers and fathers may vary and should be considered in future behaviour change programs. For instance, previous studies have shown that fathers participate in more rough-and-tumble play with their children than mothers do [35]. Thus, interventions targeting fathers may consider including rough-and-tumble play, while those targeting mothers may focus on other activities. Nonetheless, utilising the outcomes identified in this study to engage both parents in future interventions to improve children’s health behaviours may prove efficacious.

### Strengths and limitations

A main strength of this study was the large sample size for a qualitative study. In addition, the roughly equal numbers of mothers and fathers in the study, which is uncommon in research investigating young children’s behaviours adds to the study’s strength. The nature of the study allowed for two prevalent early childhood behaviours to be investigated, allowing participants to consider behaviours in context rather than in isolation, as well as aiding in understanding parents’ underlying motivations and drivers around choices of their children’s behaviours, which are additional strengths of this study. This study was however limited by the inability to determine causality due to the qualitative nature of the study However, the study aides in understanding parents’ underlying motivations and drivers around choices of their children’s behaviours.

## Conclusion

This study sought to identify parents’ perceptions of their children’s physical activity and screen time behaviours including risks and benefits of participation and potential strategies for improving behaviours. The findings from this study suggest that public health messages promoting awareness of physical activity and screen time guidelines would be beneficial for parents and early childhood professionals as key influencers of this population. Such campaigns should include clear evidence from a variety of reputable sources to raise awareness and knowledge of the evidence-based risks and benefits of physical activity and screen time during early childhood. Behaviour change interventions can draw on findings reported in this study relating to factors that hold saliency for parents and utilise those to help engage parents in interventions promoting healthy behaviours for their children.

## Additional file

Additional file 1: Interview schedule. (DOCX 14 kb)

## Abbreviations

COREQ: Consolidated criteria for reporting qualitative research
TV: television
