# Supplementary material for: Mothers’ and father’s perceptions of the risks and benefits of screen time and physical activity during early childhood: a qualitative study
Source: BMC Public Health. 2018 Nov 20;18:1271. doi: 10.1186/s12889-018-6199-6 (PMC6245522; doi:10.1186/s12889-018-6199-6)
Supplement: Supplementary file 1 — Interview schedule. (DOCX 14 kb) [file 12889_2018_6199_MOESM1_ESM.docx]

**Additional file 1: Interview Schedule**

1. Risks and benefits
   1. In what ways do you think your child might benefit from taking part in active play?
   2. What do you like about your child taking part in active play? How does it benefit you or the family?
   3. In what ways do you think active play might be detrimental to your child’s health, behaviours or development?
   4. Is there anything that concerns you about your child’s time in active play? For your child? For you? For your family?
   5. In what ways do you think your child might benefit from taking part in screen time?
   6. What do you like about your child’s screen time? How does it benefit you or the family?
   7. In what ways do you think screen time might be detrimental to your child’s health, behaviours or development?
   8. Is there anything that concerns you about your child’s screen time? For your child? For you? For your family?
2. Recommendations for young children
   1. Can you tell be about the screen time or physical activity recommendations for young children?
   2. How appropriate do you feel the recommendations are?
   3. Does ‘meeting the guidelines’ matter to you? Why/not? What does matter?
3. Changing behaviours
   1. What sorts of strategies or messages would be useful in helping families like yours increase their child’s physical activity or decrease their screen time?
   2. Who should it come from? – doctor, maternal nurse, teacher
